# Supplementary material for: The New Pharmacological Chaperones PBXs Increase α-Galactosidase A Activity in Fabry Disease Cellular Models
Source: Biomolecules. 2021 Dec 10;11(12):1856. doi: 10.3390/biom11121856 (PMC8699827; doi:10.3390/biom11121856)
Supplement: Supplementary file 1 [file biomolecules-11-01856-s001.zip › biomolecules-1464580-supplementary.pdf]

SUPPLEMENTARY MATERIALS

METHODS

Table S1: Primers used for direct mutagenesis of plasmid pR-M10-αGalA, expressing wild type *GLA* c-DNA.

PRIMER NAME	SEQUENCE (5′-3′)
**Asp170Val Fw**	ACTCCTGTGAGTGGTCTCTTTATATGTGG
**Asp170Val Rew**	CCACATATAAAGAGACCACTCACAGGAGT
**Pro205Ser Fw**	AATAAAACCTGCACAGGCTTCCCTG
**Pro205Ser Rew**	CAGGGAAGCCTGTGCAGGTTTTATT
**Gln279Arg Fw**	CTAATGACCTCCAACACATCAGCCC
**Gln279Arg Rew**	GGGCTGATGTGTTGGAGGTCATTAG
**Arg 301 Gln Fw**	TCAGCTGGAATCGGCAAGTAACTCA
**Arg301Gln Rew**	TGAGTTACTTGCCGATTCCAGCTGA

RESULTS

Table S2: Docking simulation of galactose analogues to wild type and mutated (p.Asp170A and p.Asp170V) α-GalA.

GALACTOSE											
**p.Asp170Val**				**Wild Type**				**p.Asp170Ala**			
mode	*Affinity (Kcal/mol)*	*Dist. from best mode mode*		*mode*	*Affinity (Kcal/mol)*	*Dist. from best mode mode*		*mode*	*Affinity (Kcal/mol)*	*Dist. from best mode mode*	
		RMSD m.b.	*RMSD u.b.*			*RMSD m.b.*	*RMSD u.b.*			*RMSD m.b.*	*RMSD u.b.*
1	-5.9	0.000	0.000	1	-5.7	0.000	0.000	1	-5.7	0.000	0.000
2	-5.9	1.271	2.765	2	-5.5	1.203	2.733	2	-5.4	1.183	1.183
3	-5.6	1.731	3.146	3	-5.5	1.213	1.224	3	-5.2	1.618	3.039
4	-5.2	1.520	3.816	4	-5.5	1.159	1.201	4	-4.7	1.745	3.379
5	-4.9	1.293	2.593	5	-4.6	1.364	3.794	5	-4.6	1.657	2.464
6	-4.4	1.759	4.365	6	-4.5	1.526	3.705	6	-4.6	1.226	2.718
7	-4.4	1.923	2.714	7	-4.4	1.618	2.302	7	-4.4	1.716	3.212
8	-4.4	1.287	3.549	8	-4.3	1.370	3.679	8	-4.1	1.295	2.661
9	-4.3	1.559	3.665	9	-4.2	1.756	3.813	9	-3.9	1.332	3.319
**PB48**											
**p.Asp170Val**				**Wild Type**				**p.Asp170Ala**			
mode	*Affinity (Kcal/mol)*	*Dist. from best mode mode*		*mode*	*Affinity (Kcal/mol)*	*Dist. from best mode mode*		*mode*	*Affinity (Kcal/mol)*	*Dist. from best mode mode*	
		RMSD m.b.	*RMSD u.b.*			*RMSD m.b.*	*RMSD u.b.*			*RMSD m.b.*	*RMSD u.b.*
1	-5.8	0.000	0.000	1	-5.2	0.000	0.000	1	-5.8	0.000	0.000
2	-5.5	1.578	2.790	2	-5.1	1.118	2.431	2	-5.4	1.594	1.623
3	-5.4	1.052	1.169	3	-5.0	1.261	2.427	3	-5.4	1.112	2.506
4	-5.3	1.542	2.915	4	-5.0	1.217	1.452	4	-5.0	1.820	2.973
5	-5.3	0.876	1.020	5	-4.9	1.653	2.641	5	-4.4	2.860	3.827
6	-4.6	2.411	3.071	6	-4.3	3.490	4.941	6	-4.4	1.299	1.801
7	-4.6	3.373	4.532	7	-4.2	2.292	2.809	7	-4.1	3.582	5.152
8	-4.6	1.541	1.924	8	-4.2	3.495	4.667	8	-4.1	2.360	3.055
9	-4.4	1.920	2.547	9	-4.1	1.683	2.892	9	-4.0	2.500	3.308

Supplementary Figure S1. PB48 stabilizes α-GalA when increasing temperature.

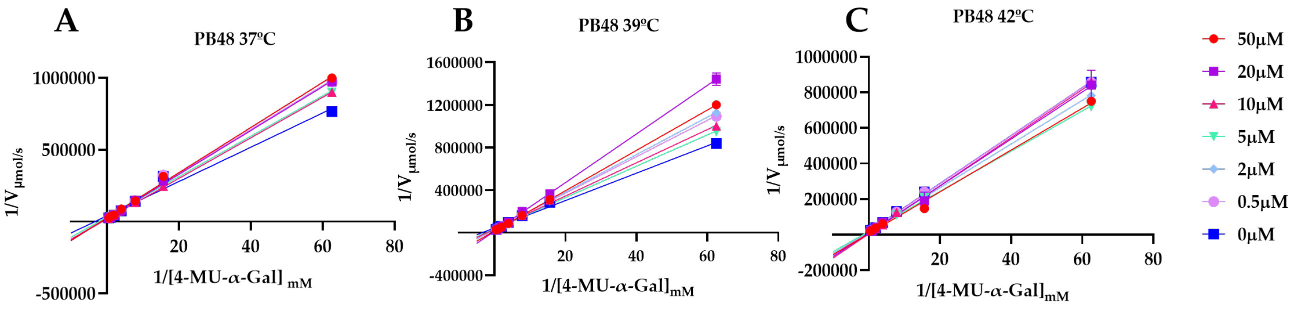

Lineweaver-Burk interpolated plots of the reciprocal values of 4MU-α-Gal substrate cleavage reaction velocity against reciprocal substrate concentration, following pre-incubation of α-GalA with PB48 (0-50 µM) at the temperature of 37 °C (A), 39 °C (B), or 42 °C (C). The reaction in presence of PB48 at the temperature of 39 °C is faster than the reaction carried out in absence of the compound for substrate concentrations higher than 0.016 mM. When the enzyme is pre-incubated with PB48 at the temperature of 42ºC the reaction is accelerated by the compound independently of substrate concentration.

Supplementary Figure S2. PB48 stabilizes α-GalA when increasing temperature at high substrate concentrations.

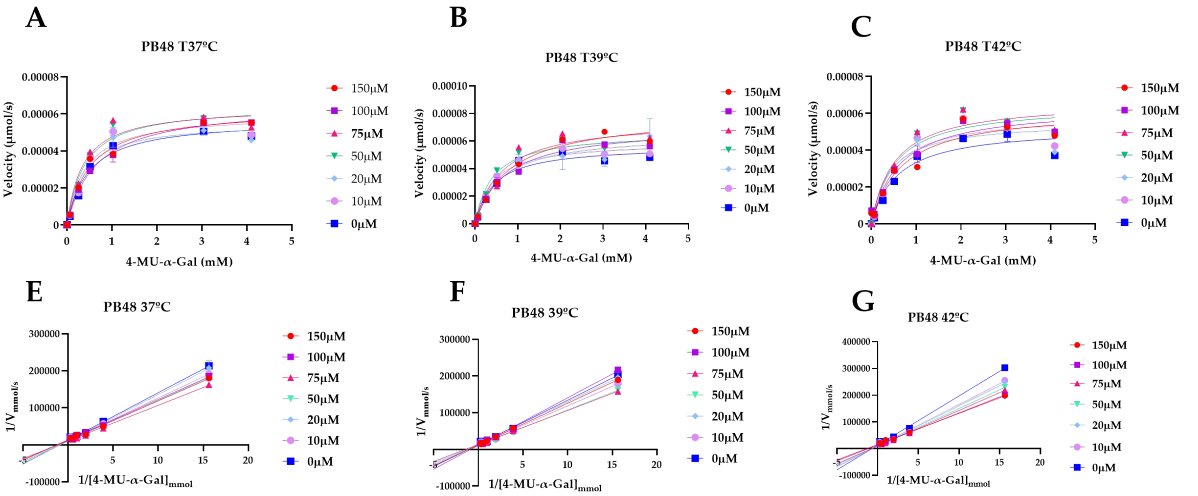

Interpolated Michaelis-Menten curves of velocity of 4MU-α-Gal substrate cleavage reaction against substrate concentration. Enzyme α-GalA was pre-incubated with PB48 (0-150μM) at the temperature of 37 °C (A), 39 °C (B), or 42 °C (C). Corresponding interpolated Lineweaver-Burk plots (E, F, G) are also shown. The velocity of the reaction in presence of PB48 at the temperature of 37°C 39°C and 42° C is accelerated, for substrate concentrations of 0.064-4.096 mM, compared to the reaction carried out in absence of PB48.

Supplementary Figure S3. Crystallographic structure of wild type α-GalA is not significantly different from the structure of p.Asp170Ala.

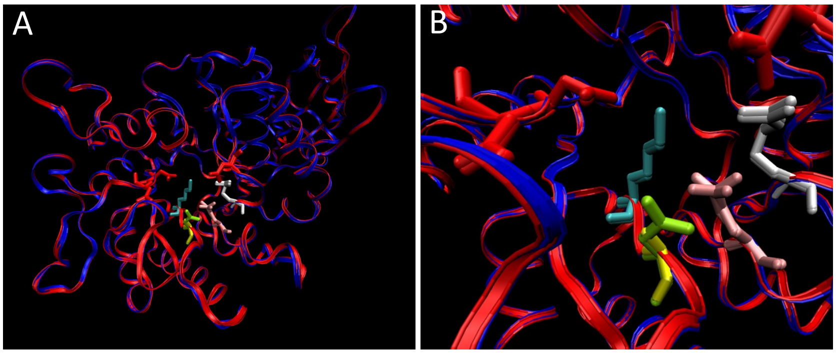

Wild type enzyme structure 3S5z (https://www.rcsb.org/structure/3s5z, blue) superimposed with variant p.Asp170Ala 3TV8 (https://www.rcsb.org/structure/3TV8, red) and relevant residues at the active site explicit in several colors. Panel A shows the whole protein and panel B focus on the active site. Both the whole protein and the active site structures are poorly affected by the replacement of the aspartic acid in position 170 with an Alanine.

Supplementary Figure S4. PB48 establishes a more extended network of contacts compared to galactose and Migalstat.

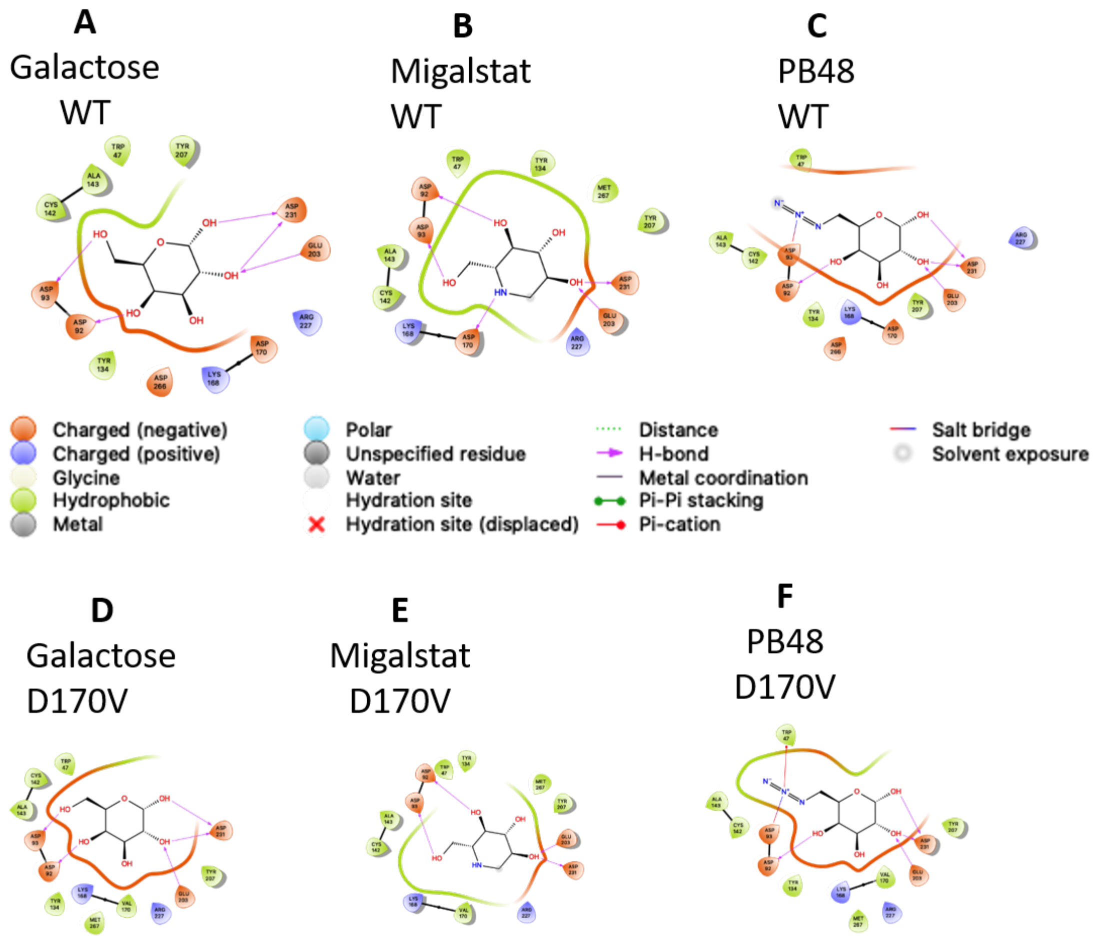

Ligand interaction diagrams for different galactose analogues on α-GalA. Panels show the interactions of galactose (A) and Miglastat (B) as observed in PDB structures 3GXP and 3GXT, respectively and modeled interaction for PB48 on wild type enzyme (C). The interactions proposed for the same ligands in complex with the p.Asp170Val mutated protein are shown in panels D-F.

Supplementary Figure S5. Cell growth curves are comparable after treatment with PB48 and PB51 and galactose

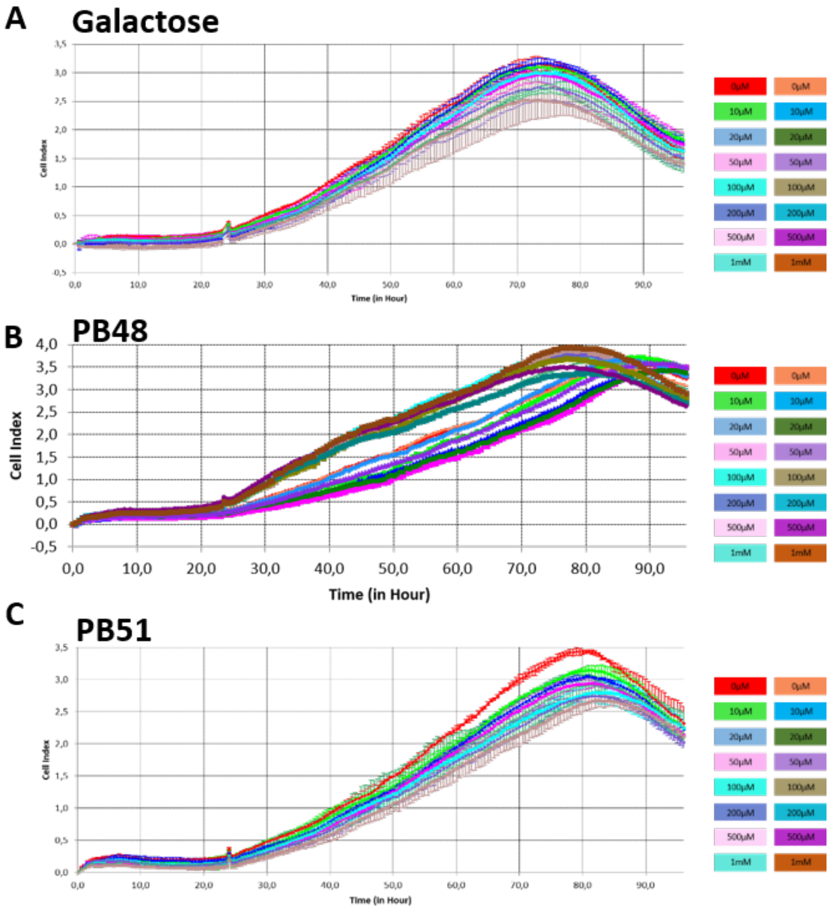

Cell growth curves (Cell index vs time) of non-transfected Hek cells treated with galactose (A), PB48 (B) and PB51 (C) in the concentration range of 0-1 mM.

Supplementary Figure S6. Hek cell growth curve and IC50after treatment with DGJ.

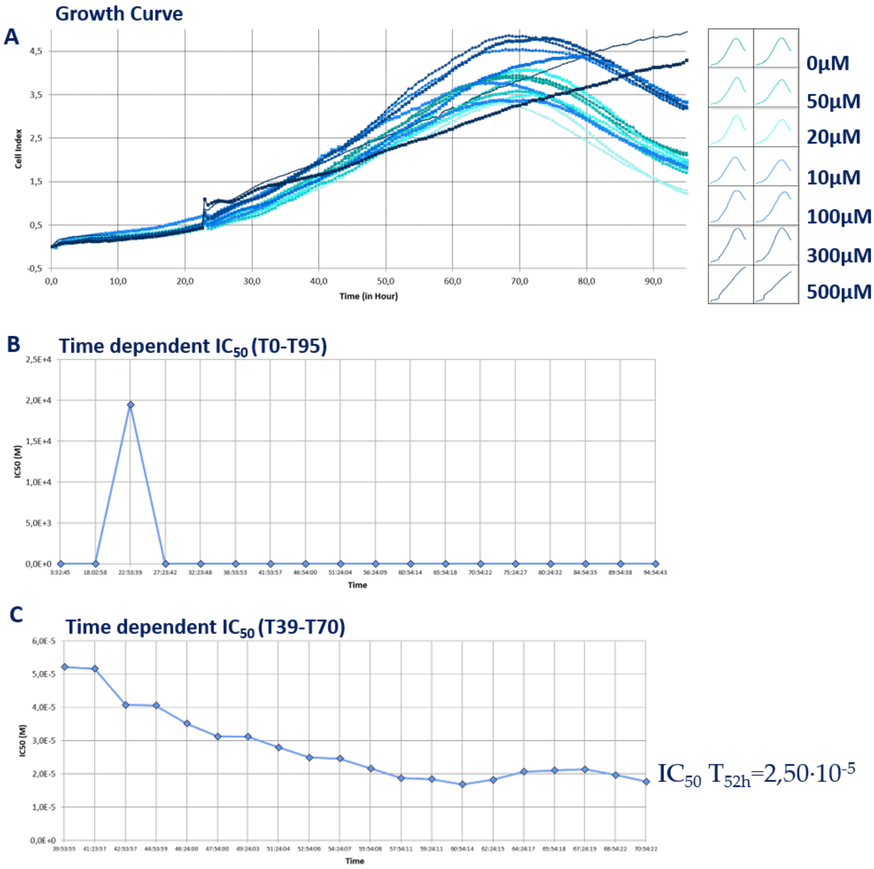

Cell growth curve (Cell index vs time, A) and plots of IC_{50} (M) versus time (hours) (B, C) of non-transfected Hek cells treated with DGJ at 0-500 µM concentration range. In B IC_{50} is represented for the whole time of acquisition, while in C it is represented in the time interval from 39 to 71 h.

Supplementary Figure S7. Effect of PB48 on α-GalA compared to galactose.

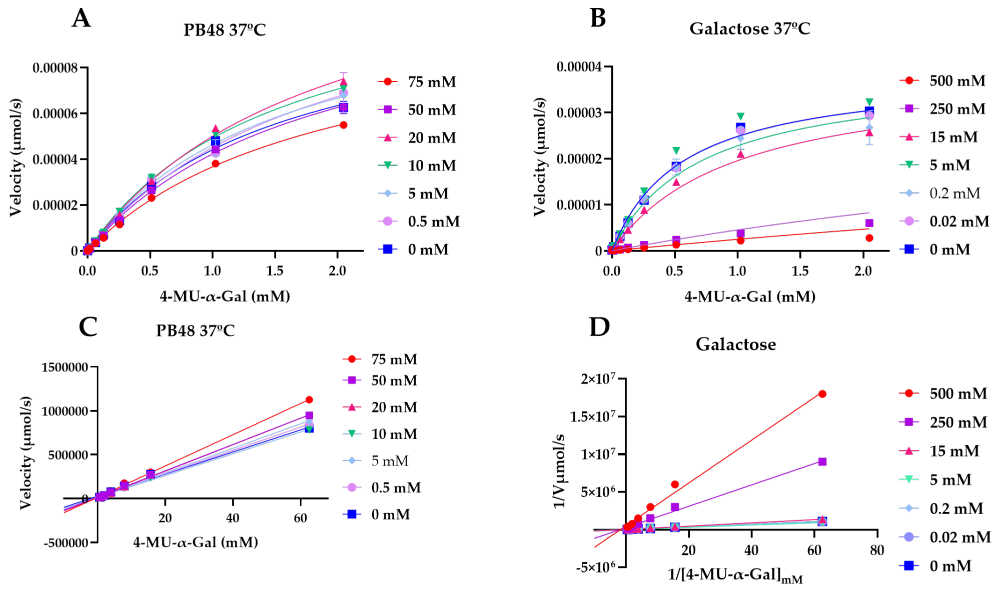

Interpolated Michaelis-Menten curves of velocity of 4MU-α-Gal substrate cleavage reaction versus substrate concentration, following pre-incubation of α-GalA with PB48 (0-75 mM) (A) or galactose (0-500 mM) (B) and the respective interpolated Lineweaver-Burk plots (C, D).

Applying the Competitive inhibitor non-linear fit (KmObs=Km*(1+[I]/Ki)Y=Vmax*X/(KmObs+X), Graph Pad Prism, analysis tool) we estimated an inhibition constant for galactose of Ki=20.11 mM ($CI_{95\%}$=16.03-25.14 mM) and PB48 Ki=255 mM ($CI_{95\%}$=167.7-472.3 mM), however the PB48 curves are not completely saturated and the reported Ki could be underestimated, thus indicating that galactose can easily replace PB48 at the active site in lysosomal conditions (pH 4.2 and 37°C).
